# Functional differentiation in the human ventromedial frontal lobe: A data‐driven parcellation

**DOI:** 10.1002/hbm.25014

**Published:** 2020-04-21

**Authors:** Henry W. Chase, Anthony A. Grace, Peter T. Fox, Mary L. Phillips, Simon B. Eickhoff

**Affiliations:** ^1^ Department of Psychiatry University of Pittsburgh School of Medicine Pittsburgh Pennsylvania USA; ^2^ Department of Neuroscience and Psychology University of Pittsburgh Pittsburgh Pennsylvania USA; ^3^ Research Imaging Institute University of Texas Health Science Center San Antonio Texas USA; ^4^ Department of Radiology University of Texas Health Science Center San Antonio Texas USA; ^5^ Department of Psychiatry University of Texas Health Science Center San Antonio Texas USA; ^6^ Research and Development Service South Texas Veterans Health Care System San Antonio Texas USA; ^7^ Institute of Systems Neuroscience, Medical Faculty Heinrich Heine University Düsseldorf Düsseldorf Germany; ^8^ Institute of Neuroscience and Medicine, Brain & Behaviour (INM‐7) Research Centre Jülich Jülich Germany

**Keywords:** connectivity‐based parcellation, functional decoding, meta‐analytic connectivity modeling, resting‐state fMRI, ventromedial frontal lobe

## Abstract

Ventromedial regions of the frontal lobe (vmFL) are thought to play a key role in decision‐making and emotional regulation. However, aspects of this area's functional organization, including the presence of a multiple subregions, their functional and anatomical connectivity, and the cross‐species homologies of these subregions with those of other species, remain poorly understood. To address this uncertainty, we employed a two‐stage parcellation of the region to identify six distinct structures within the region on the basis of data‐driven classification of functional connectivity patterns obtained using the meta‐analytic connectivity modeling (MACM) approach. From anterior to posterior, the derived subregions included two lateralized posterior regions, an intermediate posterior region, a dorsal and ventral central region, and a single anterior region. The regions were characterized further by functional connectivity derived using resting‐state fMRI and functional decoding using the Brain Map database. In general, the regions could be differentiated on the basis of different patterns of functional connectivity with canonical “default mode network” regions and/or subcortical regions such as the striatum. Together, the findings suggest the presence of functionally distinct neural structures within vmFL, consistent with data from experimental animals as well prior demonstrations of anatomical differences within the region. Detailed correspondence with the anterior cingulate, medial orbitofrontal cortex, and rostroventral prefrontal cortex, as well as specific animal homologs are discussed. The findings may suggest future directions for resolving potential functional and structural correspondence of subregions within the frontal lobe across behavioral contexts, and across mammalian species.

## 1 INTRODUCTION

The famous case of Phineas Gage (Van Horn et al., 2012), as well as more recent neuropsychological studies of patients with damage to the ventromedial regions of the frontal lobe (vmFL: Bechara, Damasio, & Damasio, 2000; Clark et al., 2008; Fellows, 2011) have helped to establish the vmFL as playing a critical role in adaptive economic and social decision‐making in humans (Delgado et al., 2016). Studies of human lesion patients have, in general, only limited capacity to provide finely resolved anatomical detail regarding the precise organization within this structure that underlies the profound deficits that can be observed (Mah, Husain, Rees, & Nachev, 2014, but see Glascher et al., 2012; Manohar & Husain, 2016). Thus, the extent to which different syndromes and dimensions of psychiatric symptomatology are associated with alteration of particular subregions of the vmFL remains uncertain.

Nevertheless, differentiation of function within the vmFL is anticipated by at least two separate lines of evidence. First, confined lesions of this region in rodents and monkeys can reveal distinct patterns of deficit on flexible decision‐making tasks, depending on the precise location of the lesion and the task employed (Chudasama & Robbins, 2003; Killcross & Coutureau, 2003; Rudebeck, Bannerman, & Rushworth, 2008; Rudebeck & Murray, 2011). Consequently, the concept of the vmFL as a unitary neuroanatomical region is challenged by these experiments, and may refer to several independent but interacting subregions. Further evidence that the vmFL is not a single entity is a result of neuroanatomical evidence delineating granular and agranular subregions within the vmFL (Barbas & Garcia‐Cabezas, 2016; Henssen et al., 2016; Wise, 2008). Presupposing that functional differences may be carried by of anatomical differences (Eickhoff, Constable, & Yeo, 2018), there are significant cytoarchitectonic differences that might subserve the functional differences described earlier. For example, Mackey and Petrides (Mackey & Petrides, 2014) describe a gradient of increasingly developed, granular cortex from posterior (i.e., subgenual and pregenual regions of the cingulate cortex) to anterior (i.e., rostromedial prefrontal cortex) regions, with more posterior regions showing a more primitive, agranular cytoarchitecture. The vmFL also shows differential patterns of anatomical connectivity, across its structure, with distal regions including the striatum, insula, thalamus, hypothalamus, hippocampus, amygdala, and other temporal lobe structures (Heilbronner, Rodriguez‐Romaguera, Quirk, Groenewegen, & Haber, 2016; Hoover & Vertes, 2007; Reppucci & Petrovich, 2016; Saleem, Kondo, & Price, 2008; Vertes, 2004).

These separate lines of evidence thus appear to favor separate functional and anatomical subregions within the human vmFL. Briefly, these subregions may tentatively include subgenual and pregenual regions of the anterior cingulate cortex (ACC), the medial orbitofrontal cortex (OFC), and parts of the rostromedial prefrontal cortex corresponding to Brodmann Area 10 (Ongur, Ferry, & Price, 2003). There remains relatively little in vivo evidence for functional differentiation along these lines in humans, however, and functional similarities among these regions can be emphasized (Delgado et al., 2016). Moreover, cross‐species homologies are potentially more distant in the frontal lobe compared to other neural structures (Wise, 2008), underscoring the need for supporting evidence in humans to supplement work in experimental animals.

In the present study, we employed a parcellation algorithm which has been applied to distinguishing clusters of similar brain (co‐)activation patterns stored within the BrainMap database (P. T. Fox & Lancaster, 2002). Examining patterns of co‐activation across the brain is a well‐established approach to assessing functional connectivity (Eickhoff et al., 2011). Thus, the combination of co‐activation maps with data‐driven clustering algorithms offers the possibility of distinguishing subregions within a larger region of interest based on interaction with different distal regions and/or networks. This method has been applied to identify distinct regions within the dorsolateral prefrontal cortex (Cieslik et al., 2013), the posterior cingulate (Bzdok et al., 2015), the amygdala (Bzdok, Laird, Zilles, Fox, & Eickhoff, 2013) and the subiculum (Chase et al., 2015), for example. In several cases, the subregions that have been identified are regions with known anatomical and functional differences: for example, Bzdok et al. found a parcellation of the amygdala that corresponded well with the classic tripartite (superficial, basolateral, and centromedian) model of the region (Bzdok et al., 2013). In the case of the vmFL, there is already some evidence that such a parcellation of distinct regions is possible using co‐activation maps: Clithero and Rangel found evidence for three distinct clusters of activation within the vmFL when examining fMRI literature on value encoding in this region (Clithero & Rangel, 2014): see also (Hiser & Koenigs, 2018) for another meta‐analytic approach). In contrast to these previous parcellation studies, a novel aspect of the present work, from a methodological point of view, is the use of a two‐stage parcellation to address the indeterminacy of the boundaries of the vmFL—the lateral and dorsal extents in particular. First, a rather general vmFL region of interest was drawn by hand which was used for the initial parcellation. A second parcellation was performed on the best‐resolved subregions within the ventral and medial regions of the frontal lobe, with ambiguous, peripheral regions being omitted from this analysis. It was hoped that this would lead to a parcellation which effectively balanced completeness and focus on the vmFL.

We expected to distinguish regions corresponding to the human homologs of the infralimbic (IL) and prelimbic (PL) cortices, which are likely to correspond to Brodmann area 25 and 32 in humans (Azuma & Chiba, 1996; Heilbronner et al., 2016; Room, Russchen, Groenewegen, & Lohman, 1985; Takagishi & Chiba, 1991). We also expected to identify regions corresponding to the medial OFC (11, 14) and posterior regions of Brodmann Area 10 (Mackey & Petrides, 2014; Ongur et al., 2003; Wise, 2008). The frontal pole itself was not a primary focus of the study, and has been previously examined in the context of other anatomical studies (Bludau et al., 2014; Ray et al., 2015). The functional connectivity of the emerging subregions was expected to correspond to known anatomical relationships with distal neural regions, including the posterior cingulate cortex (PCC), amygdala, hippocampus, temporal lobe, midbrain, ventral striatum, and thalamus. Although these expectations were grounded in empirical data from rodents and nonhuman primates, the precise location and functional characteristics of the hypothesized regions has not, to the best of our knowledge, been confirmed in humans despite the fact that in vivo evidence of functional and anatomical differentiation has been obtained (Clithero & Rangel, 2014; Samara et al., 2017). Furthermore, recent work suggests that different brain states may influence the nature of parcellation, and thus different methods cannot be assumed to yield identical subregions (Kahnt & Tobler, 2017; Salehi et al., 2020). The Meta‐analytic connectivity modeling (MACM) parcellation approach using the BrainMap database might therefore provide complementary insights into the context‐specificity of parcellation within the vmFL.

MACM was used to estimate functional connectivity, and was used as the basic dependent measure for the parcellation. Seed‐based connectivity using resting‐state fMRI (rsfMRI) was used to characterize the functional connectivity of the resulting subregions further. RsfMRI contributes an important inferential component to the present work: the MACM maps for each subregion were used to define the subregion via the parcellation, and thus estimates of connectivity are thus somewhat circularly defined. RsfMRI can be used to provide confirmation that similar functional connectivity can also be observed using an independent data set and methodology.

Finally, we aimed to characterize the neurofunctional properties of the regions. To the best of our knowledge, a comprehensive examination of the functional profile of the activation properties of the region has not been conducted across a variety of psychological tasks (i.e., the types of paradigm and psychological domain that activate the region). Using the BrainMap database, we examined the paradigms and psychological domains that activated the identified subregions of the vmFL, and also confirmed the capacity of activation within the vmFL to be decoded in terms of a particular paradigm or domain.

## 2 METHODS

### 2.1 ROI definition and methods overview

The demarcation of the vmFL ROI used for clustering was centered on a roughly defined region (Figure S4), based largely on the medial OFC regions defined within the automated anatomical labeling template (Tzourio‐Mazoyer et al., 2002). However, this region was expanded considerably by drawing a wider volume around the predefined OFC regions using the Marina tool (Walter et al., 2003). This expanded region comprised the entire anterior–posterior dimension of the ventral frontal cortex, that is, from just anterior of the ventral striatum to the entire frontal pole, and extended dorsally just above the genu. We took this approach in order to avoid dependence on any one particular anatomical scheme, and to be as inclusive as possible. As this definition was relatively arbitrary, this region formed the basis for an initial parcellation which would define a focus for a subsequent parcellation, mostly by removing the lateral, anterior (i.e., frontal polar) and dorsal edges of the region (Figure S5). The second parcellation would then be conducted on the clusters derived from this initial parcellation which most clearly reflected the ventromedial aspect of the frontal lobe. This second parcellation is the main focus of the analysis: we characterized the subregions derived from this second parcellation in terms of their co‐activation patterns, resting‐state functional connectivity, and BrainMap‐database derived activation properties.

### 2.2 Meta‐analytic connectivity mapping (MACM) and connectivity‐based parcellation

Parcellation involved the MACM‐based approach employed in previous studies (Bzdok et al., 2013; Cieslik et al., 2013). Briefly, the MACM approach involves computing the co‐occurrence of significant activations across studies within each voxel within the vmFL volume of interest (VOI). Data from the BrainMap database were used (www.brainmap.org; P. T. Fox & Lancaster, 2002; Laird et al., 2011), registered into Montreal Neurological Institute (MNI) space. Given the sparsity of voxel‐wise activation, experiments were pooled within the vicinity of each seed voxel using a spatial filter of different sizes. A subsequent coordinate‐based meta‐analysis was performed on the retrieved experiments, generating a brain‐wide co‐occurrence of activation profile of a given seed voxel, for each filter size (37). The brain‐wide pattern of co‐occurrence for each individual seed voxel was computed by activation likelihood estimation (ALE; Eickhoff, Bzdok, Laird, Kurth, & Fox, 2012; Turkeltaub, Eden, Jones, & Zeffiro, 2002) meta‐analysis over the experiments that were associated with that particular voxel by the pooling procedure outlined earlier. However, no thresholding was performed at this stage, with the goal to obtain whole‐brain map of co‐occurrence probabilities for each seed voxel, to be used as a basis for parcellation of each VOI.

Following our previous studies (Bzdok et al., 2013; Cieslik et al., 2013), we used *K*‐means clustering (MATLAB, Mathworks) to parcellate each VOI, using *K* = 2–10 for the first and *K* = 2–11 for the second. *K*‐means clustering was performed on the unthresholded brain‐wide co‐occurrence profiles for all seed voxels: specifically, the NS × NT co‐occurrence matrix, where NS denotes the number of seed voxels in each VOI and NT the number of target voxels in the (downsampled) reference brain volume. The distance measure used for the *K*‐means clustering was one minus the correlation between the co‐occurrence patterns of seed voxels defined earlier (correlation distance). Importantly, maps of co‐occurrence of activations were computed for each of the 37 different spatial filter sizes, and the *K*‐means parcellation was performed for each filter size independently, yielding (*K* clusters) × 37 (filter size) independent cluster solutions (Clos, Amunts, Laird, Fox, & Eickhoff, 2013). Replications of each parcellation were performed for each VOI using random initial conditions (centroids), to avoid local minima.

### 2.3 Selection of optimal filter range and number of clusters

Our approach to selecting the optimal solution of *K*‐means clustering was to examine the properties of these various solutions and establish the most stable range of filter sizes. This prevented a combinatorial expansion of possible solutions, and avoided the requirement of averaging across filter sizes (Bzdok et al., 2013; Cieslik et al., 2013). We implemented a two‐step procedure that involved a decision on those filter sizes to be included in the final analysis and subsequently a decision on the optimal cluster solution. In the first step, we examined the consistency of the cluster assignment for the individual voxels across the cluster solutions of the co‐occurrence maps performed at different filter sizes. We selected a filter range with the lowest number of deviants, that is, number of voxels that were assigned differently compared with the solution from the majority of filters. In other words, we identified those filter sizes which produced solutions most similar to the consensus‐solution across all filter sizes. For example, the proportion of deviants for the second parcellation is illustrated in Figure S1; this shows the borders of the filter range to be used for subsequent steps was based on the *Z*‐scores of the number of deviants.

The second step was to determine the optimal solution of *K* within the restricted filter range of filter sizes. We considered five criteria representing the characteristics of the cluster solutions, reflecting topological, information‐theoretic, and cluster separation properties (Figures S2 and S3). First, misclassified voxels (deviants) were examined as a topological criterion, with optimal *K* parcellations being those in which the percentage of deviants was not significantly increased compared to the *K* − 1 solution and but where the *K* + 1 was associated with significantly increased deviants (Bzdok et al., 2015). Second, the variation of information (VI) metric was employed as an information‐theoretic criterion to assess the similarity of cluster assignments for each filter size between the current solution and the neighboring (*K* − 1 and *K* + 1) solutions (Meila, 2007). Third, the silhouette value averaged across voxels for each filter size was considered a cluster separation criterion. Fourth, we assessed the percentage of voxels not related to the dominant parent cluster compared with the *K* − 1 solution as a second topological criterion. This measure corresponds to the percentage voxels that are not present in hierarchy, *K*, compared with the previous *K* − 1 solution, and is related to the hierarchy index (Kahnt, Chang, Park, Heinzle, & Haynes, 2012). Finally, the change in inter‐ versus intra‐cluster distance ratio was computed (Chang, Kenney, Loucks, Poletto, & Ludlow, 2009) as a second cluster separation criterion. This ratio is the first derivative of the ratio between the average distance of a given voxel to its own cluster center and the average distance between the cluster centers.

### 2.4 Visualization of the best cluster solution

Voxels which were located in the gray matter, and were hierarchically and spatially consistent, were considered for subsequent analyses. Multidimensional scaling (MDS) was used to visualize the 2‐dimensional cluster separation. We computed the NS × NS correlation distance matrix (Section 2.3) for each of the filter sizes. Next, MDS was performed on the eigenimage of the resulting correlation distance matrixes, with Sammon's nonlinear mapping being used as the goodness‐of‐fit criterion. Finally, the locations of the clusters were mapped back on the brain, taking the mode across filter sizes.

### 2.5 Functional connectivity analysis: Task‐based and resting‐state

Further analyses were conducted to characterize the subregions resulting from the second vmFL parcellation, which reflected the core region of interest for the present work. First, meta‐analytic connectivity modeling (MACM) was employed. For this, all experiments in the BrainMap database that featured at least one focus of activation in a particular subregion were compiled, and convolved with a 3D Gaussian as described in Section 2.2. However, now conventional inference was performed with reference to a null distribution reflecting a random spatial association between experiments with a fixed within‐experiment distribution of foci (Eickhoff et al., 2009). A nonparametric *p*‐value based on the proportion of equal or higher random values than the null distribution was thereby obtained for each voxel (Eickhoff et al., 2012), and these were transformed into *Z*‐scores and thresholded at a cluster‐level family‐wise error (FWE) rate‐corrected threshold of *p* < .05 (cluster‐forming threshold at voxel‐level *p* < .001).

We tested for differences in co‐occurrence patterns between all pairs of clusters by performing MACM separately on the experiments associated with either cluster, and then computing the voxel‐wise difference between the ensuing ALE maps. All experiments contributing to the two contrasted clusters were pooled and randomly divided into two groups of the same size as the two original sets of experiments defined by activation in the first or second cluster (Eickhoff et al., 2011). ALE‐scores for these two randomly assembled groups were calculated and the difference between these ALE‐scores was recorded for each voxel in the brain. Repeating this process 10,000 times then yielded a null distribution of differences in ALE‐scores between the MACM analyses of the two clusters. The “true” difference in ALE scores was then tested against this null distribution yielding a posterior probability that the true difference was not due to random noise in an exchangeable set of labels based on the proportion of lower differences in the random exchange. The resulting probability values were then thresholded at *p* > .95 (95% chance for true difference) and inclusively masked by the respective main effects, that is, the significant effects in the MACM for the particular cluster. To simplify the analysis and reduce the number of comparisons, we computed a conjunction map of the contrasts of a given subregion with all others (e.g., Clusters 1 vs. 2–6). This would identify regions which were preferentially connected to the subregion compared to all other subregions.

In addition, we also delineated the task independent resting‐state functional connectivity pattern of each cluster from the second parcellation. RsfMRI images of 196 healthy volunteers (mean age 39.8 ± 15.1 years; 76 males) from the enhanced Nathan Kline Institute (NKI)/Rockland sample were obtained through the 1,000 Functional Connectomes Project (www.nitrc.org/projects/fcon_1000/). Acquisition parameters and preprocessing of these images has been described in previous work (Chase et al., 2015). Briefly, 404 echo‐planar images (EPIs) were acquired on a Siemens Trio 3T scanner, using a TR of 1.4 s. Preprocessing involved realignment and normalization using the “unified segmentation” approach into MNI space, followed by spatial smoothing with a 5 mm Gaussian kernel. Nuisance correction was then performed using motion parameters and their first derivatives, and mean cerebrospinal fluid, gray, white matter time series. Following nuisance correction, the time series were band pass filtered between 0.01 and 0.08 Hz. The connectivity‐based parcellation (CBP)‐derived clusters from the second parcellation were used as seeds for the resting‐state analysis. Linear (Pearson) correlation coefficients between the time series of the seed regions and all other gray matter voxels in the brain were computed to quantify rsfMRI connectivity. These voxel‐wise correlation coefficients were then transformed into Fisher's *Z*‐scores and tested for consistency in a flexible factorial model across subjects.

We used these maps for two analyses. First, we investigated the similarity between the MACM and resting‐state analyses: rsfMRI *Z*‐score maps were masked using the thresholded maps from the MACM analysis: inference was performed only within the regions identified as co‐activated by a MACM analysis using the corresponding subregion as a seed. A cluster was reported as significant in Table 2 (positive/negative: far right‐hand column) if an FWE‐corrected peak threshold of *p* < .05 was reached (corrected for voxels within the MACM mask rather than the whole brain). Second, we examined whole‐brain connectivity for each subregion using a similar strategy for the MACM contrast maps, that is, calculate a given subregion's positive functional connectivity compared to all others. The contrast was weighted so that the target region was coded as 5 and the other regions were coded as −1. Post hoc analyses were performed to distinguish subregions with similar connectivity. The standard SPM8 implementations were used including appropriate nonsphericity correction, and correction for age and gender. These analyses were thresholded at *p* < .05 (FWE cluster‐corrected; cluster‐forming threshold at voxel‐level *p* < .001).

### 2.6 Functional characterization: Meta‐data

The functional characterization of the CBP‐derived clusters was based on the “Behavioral Domain” and “Paradigm Class” meta‐data categories available for each neuroimaging experiment included in the BrainMap database. Behavioral domains include the main categories cognition, action, perception, emotion, and interoception, as well as their related sub‐categories. Paradigm classes categorize the specific task employed (see http://brainmap.org/scribe/ for the complete BrainMap taxonomy).

In a first step, we determined the individual functional profile of the six CBP‐derived clusters by using forward and reverse inference (Bzdok et al., 2013; Cieslik et al., 2013). Forward inference is the probability of observing activity in a brain region given knowledge of the psychological process, whereas reverse inference is the probability of a psychological process being present given knowledge of activation in a particular brain region. In the forward inference approach, a cluster's functional profile was determined by identifying taxonomic labels, for which the probability of finding activation in the respective cluster was significantly higher than the overall chance (across the entire database) of finding activation in that particular cluster. Significance was established using a binomial test (*p* < .05, corrected for multiple comparisons with reference to the false discovery rate [FDR]). Thus, we tested whether the conditional probability of activation given a particular label (P[Activation|Task]) was higher than the base rate probability of activating a given subregion per se (P[Activation]). In the reverse inference approach, a cluster's functional profile was determined by identifying the most likely behavioral domains and paradigm classes given activation in a particular subregion. This likelihood P(Task|Activation) can be derived from P(Activation|Task) as well as P(Task) and P(Activation) using Bayes' rule. Significance was then assessed by means of a chi‐square test (*p* < .05, FDR corrected).

## 3 RESULTS

### 3.1 Connectivity‐based parcellation

The clustering analysis proceeded in two stages. The initial vmFL ROI was deliberately drawn to be large (Figure S4), and had relatively few anatomical constraints defining its shape. After the first parcellation, a nine‐cluster solution was chosen (Figure S5). This was supported in the following ways: a significant increase in VI from 9 to 10, but not 8 to 9; a significant increase in silhouette from 8 to 9, but not 9 to 10; VI across clusters being very low at 9 but high going to 10, indicating that from 8 to 9 there is good consistency, but little consistency from 9 to 10; change in inter/intra‐cluster distance identified up to 9, which is a local maximum, that is, separation becomes much better when going to 9 but improves little going up to 10; a decline in misclassified voxels from 8 to 9 but a significant increase from 9 to 10.

Of the nine subregions, we focused on four as reflecting the vmFL (numbers 2, 4, 7, and 8) and excluded the remaining five. The selected clusters occupied ventral and medial regions of the frontal lobe: in particular, the medial width of selected regions was similar to anatomical definitions of the medial OFC (Henssen et al., 2016). Of the excluded clusters, Regions 1 and 3 were not fully resolved, and corresponded to regions that might be distinct from the vmFL (anterior cingulate and frontal pole). Interesting, the frontal pole cluster overlapped clearly with an anatomically defined ROI of the frontal pole (Bludau et al., 2014). The remaining clusters (5, 6, and 9) were also not fully resolved, appearing on the edges of the initial vmFL ROI (Figure S5).

**FIGURE 1:**
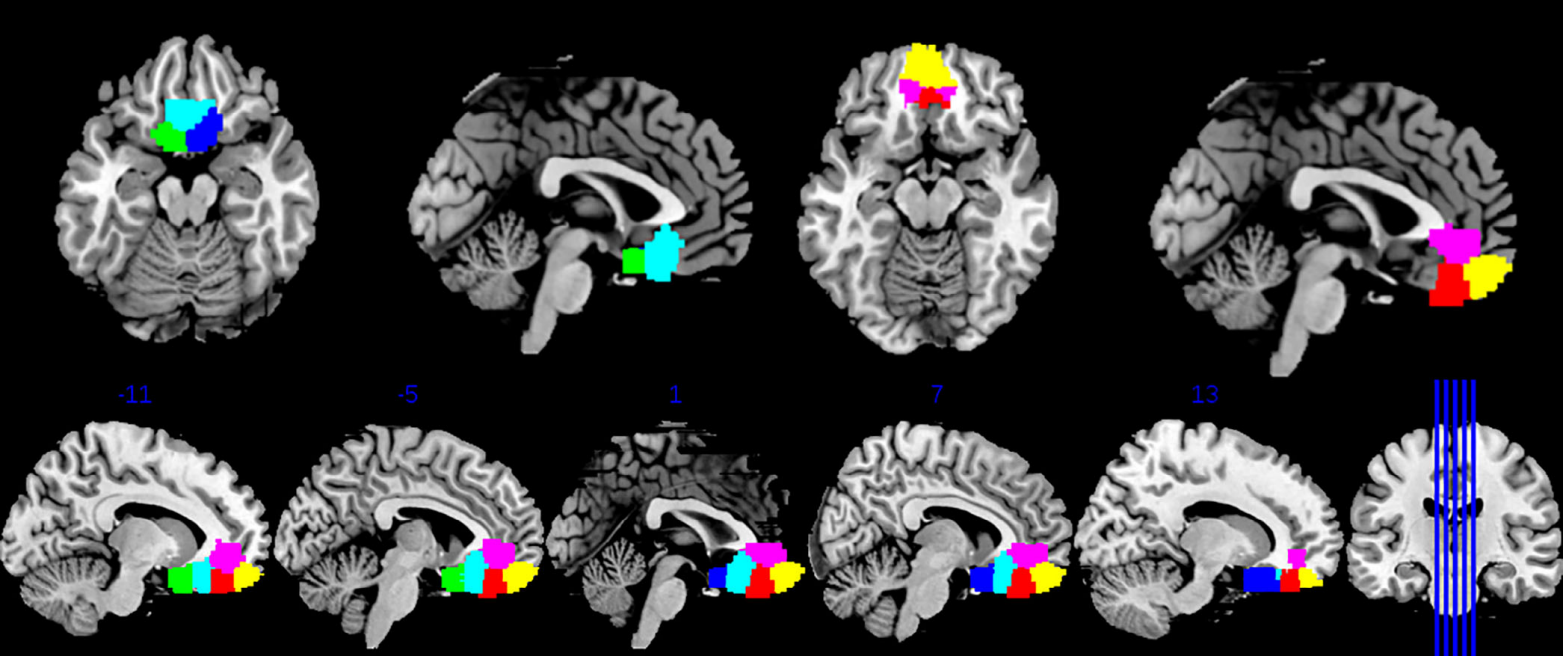
Locations of the six‐cluster solution within the vmFL (clusters color‐coded as follows: 1 = blue, 4 = cyan, 6 = green; 2 = yellow, 3 = magenta, 5 = red). vmFL, ventromedial regions of the frontal lobe

We then performed a second parcellation on the four vmFL subregions from the initial parcellation, which yielded a six‐cluster solution (Figure 1). This parcellation was assessed using the same measures as before, and supported in the following ways: silhouette—a significant increase from 5 to 6 but a decrease from 6 to 7; VI across clusters—very low up to 6 but high from 7 onward, indicating that from 5 to 6 there is good consistency, but little from 6 to 7; change in inter/intra‐cluster distance shows that 6 is a local maximum, that is, separation becomes much better when going to 6 but improves little going to 7. VI across filter sizes was uninformative, as it increased similarly across cluster solutions. However, misclassified voxels did not support the six‐cluster solution, since there was an increase from 5 to 6 but none from 6 to 7. Thus, on balance, a six‐cluster solution was preferred, if less decisively than the first parcellation. For comparison purposes, we have included figures of the nonpreferred cluster solutions in supplementary information (Clusters 3–5/7–8; Figure S6).

The six clusters which were derived are displayed in Figure 1, and include two subgenual clusters (1 and 6), a posterior cluster (4), two central clusters (dorsal [3] and ventral [5]), and one anterior cluster (2). We examined the overlap of these clusters with anatomical parcellations of OFC (Henssen et al., 2016; Mackey & Petrides, 2014); see Table 1). Neither of the anatomical parcellations cleanly mapped onto the six CBP‐derived clusters: rather, the six CBP clusters were made up of very roughly similar ratios of cingulate (e.g., area 24/32) and orbitofrontal (e.g., Fo/Fp or areas 11/14) anatomical labels derived from both templates.

**TABLE 1:** Overlap of the six clusters with the JuBrain template and the Mackey and Petrides ventromedial atlas (percentages describe proportions of cluster within given anatomical label; all overlap >2% reported)

	Anatomy toolbox (JuBrain)	Mackey & Petrides, 2014
Cluster 1	42.8% Fo2; 11.3% Fo3; 5.3% area 25; 3.2% area s24 (all right)	35% right 14c 24.5% right 14r 10.5% right 25 5.7% right 32
Cluster 2	15.1% right Fo1; 14.7% left Fp2; 11.7% left Fo1; 11.6% left Fp1	8.8% left 11 m 13.4% right 11 m 6.5% left 14 m 7.5% right 14 m
Cluster 3	22.5% left area p32; 16.9% right area p32; 10.1% left area p24ab; 9.0% left area s32	31.2% left 14 m 27.9% right 14 m 3.3% left 24 3.2% right 24 9.6% left 32 8.0% right 32
Cluster 4	22.3% left area Fo2; 15.1% left area s24; 15.0% right area Fo2; 11.3% right area s24	3.6% left 14c 2.8% right 14c 19.3% left 14 m 18.0% right 14 m 12.2% left 14r 11.6% right 14r 2.1% left 24 7.8% left 32 6.4% right 32
Cluster 5	32.8% right area Fo1; 29.3% left area Fo1; 4.6% left area s32; 4.1% right area s32	9.3% left 11 m 10.6% right 11 m 11.6% left 14 m 13.3% right 14 m 3.4% left 14r 5.8% right 14r 12.5% left 14r′ 18.8% right 14r′
Cluster 6	36.1% Fo2; 7.9% area 25; 4.6% area Fo3; 2.8% area 33 (all left)	45.0% left 14c 3.4% left 14r 13.7% left 25 5.5% left 32

### 3.2 Functional connectivity (MACM/rsfMRI)

**FIGURE 2:**
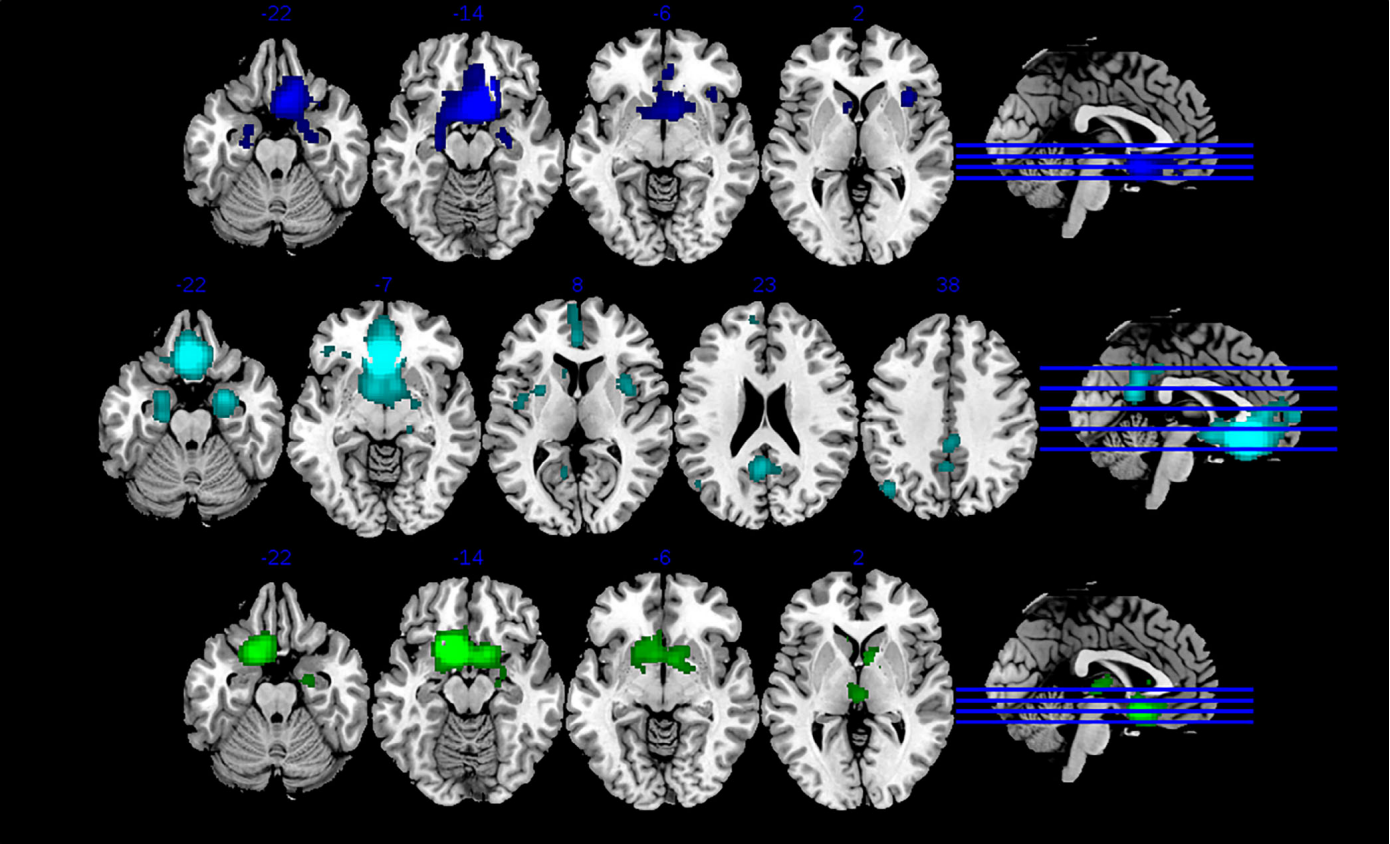
MACM plots for each of the posterior subregions. Clusters color‐coded as previously—top to bottom: 1 = blue, 4 = cyan, 6 = green. MACM, meta‐analytic connectivity modeling

**FIGURE 3:**
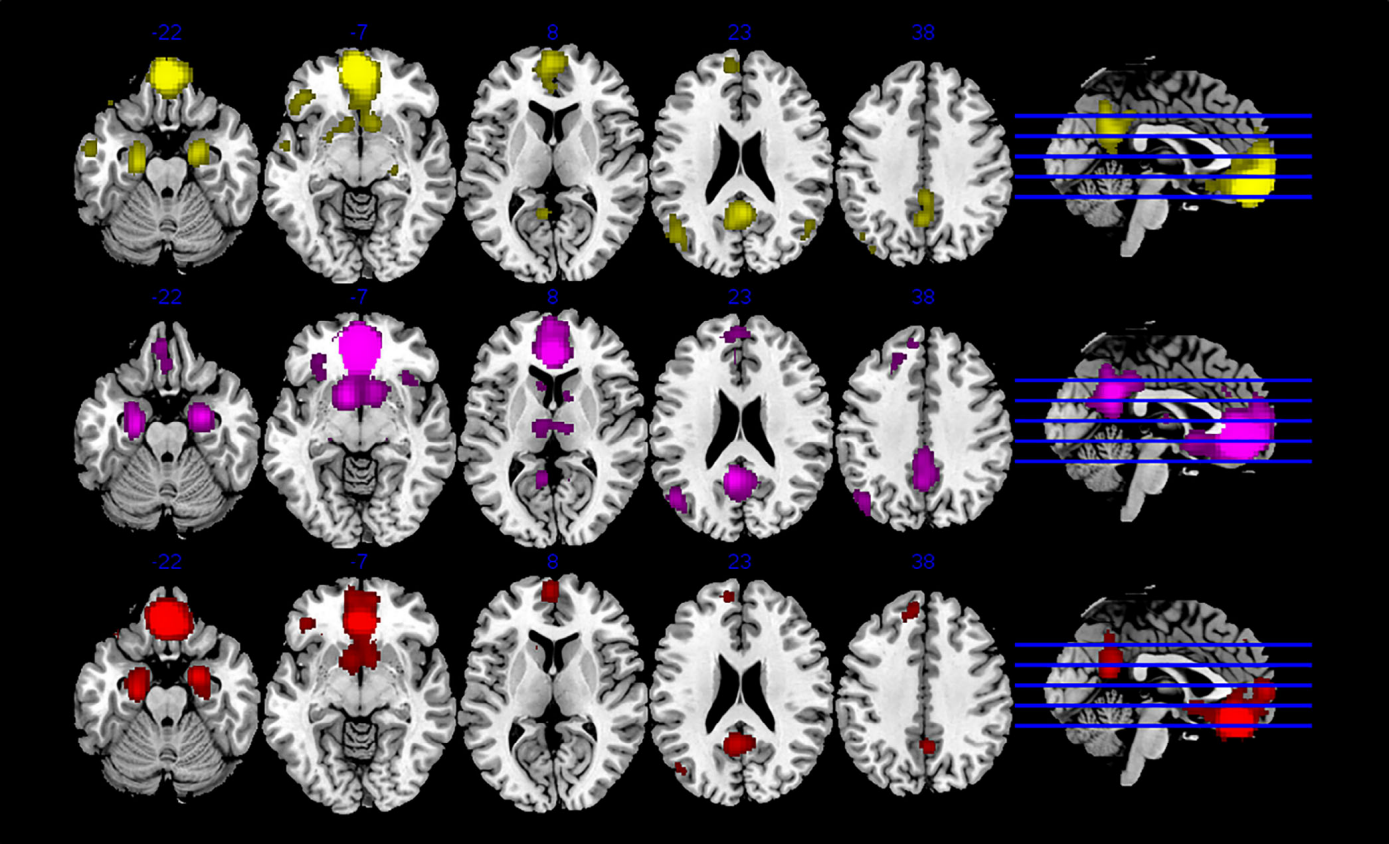
MACM plots for each of the anterior subregions. Clusters color‐coded as previously—top to bottom: 2 = yellow, 3 = magenta, 5 = red. MACM, meta‐analytic connectivity modeling

We examined the connectivity of the six clusters using MACM and resting fMRI (Figures 2 and 3; Table 2). Several broad patterns emerged. As the vmFL is part of the default mode network (DMN; Raichle et al., 2001), many of the clusters showed evidence of functional connectivity with posterior cingulate, temporal and parietal regions canonically associated with this network. In addition, regions with strong anatomical connectivity with the vmFL, including the ventral striatum, amygdala, hippocampus, and thalamus, also showed strong functional connectivity. Specifically, the most posterior, subgenual Clusters 1 and 6 were connected to proximal regions of the vmFL, and the ventral striatum and amygdala, the right insula (Cluster 1 only), the thalamus (Cluster 6 only). The other posterior cluster (4) was connected to a number of DMN regions including the PCC and the superior frontal gyrus (SFG), and also the insula, left OFC, and the ventral striatum, amygdala, and hippocampus. The two central clusters (3 and 5) were similarly connected to the DMN, ventral striatum, amygdala, hippocampus, the thalamus (Cluster 3 only), and left OFC (Cluster 5 only). Finally, the most anterior cluster showed connectivity with the DMN, the left ventrolateral prefrontal cortex (VLPFC), the amygdala and hippocampus, and the left middle temporal gyrus. In general, patterns of functional connectivity as estimated using MACM overlapped well with those using rsfMRI: Table 2 describes the present of significant (small volume corrected) rsfMRI functional connectivity within the regions specified by MACM. There was only one clear contradiction to this pattern, aside from a few null findings: Cluster 4 showed (positive) functional connectivity with the right dorsal insula using MACM, but anti‐correlation using rsfMRI.

**TABLE 2:** MACM coordinates for all six clusters, as well as the presence of overlap with significant rsfMRI connectivity in the cluster

	Cluster size	Peaks	RsfMRI
Cluster 1 (right sgACC)			
Large cluster including: Ventromedial prefrontal cortex Bilateral amygdala (LB/SF) Bilateral hippocampus (CA) Bilateral medial ventral striatum Left caudate	3,556	12, 12, –14 −20, −6, −16 6, 38, –10 30, –14, –16 −22, −14, −14	Positive including: vmFL Medial VS Right Amyg Left HC
Right anterior insula	211	36, 18, 2	Positive
Cluster 2 (anterior medial)			
Large cluster including: Anterior vmFL Frontal pole/dorsomedial PFC Left amygdala (LB) Left hippocampus (CA) Bilateral ventral striatum/anterior caudate	5,188	−2, 50, –14 −20, −16, –20 10, 14, –10 −2, 40, 4 −10, 12, –6 −8, 56, 24	Positive including: vmFL Frontal pole HC/Amyg Medial VS
Precuneus/posterior cingulate	1,468	2, –52, 26 2, –44, 36 −6, −54, 10 2, –30, 46	Positive
Right hippocampus (CA)/right amygdala (LB/SF)	651	24, –6, −20 28, –18, −16	Positive
Left angular gyrus (PGp/PGa)	521	−48, −70, 28 −42, −78, 32 −50, −58, 26	Positive
Left ventrolateral PFC	396	−41, 34, –8 −42, 30, –18 −48, 26, –6 −48, 26, –12	Positive
Left middle/superior temporal gyrus	284	−58, −6, −18 −56, −2, −6	Positive
Right middle temporal gyrus (PGp/PGa)	145	50, –68, 18 50, 62, 22	Positive
Cluster 3 (central dorsal)			
Large cluster including: Ventromedial PFC Bilateral medial ventral striatum/anterior caudate Bilateral amygdala Bilateral hippocampus Left central OFC, anterior insula	8,617	0, 48, –6 −10, 10, –6 −24, −14, –20 26, –6, −20 12, 10, –6 10, 14, –8	Positive (all regions)
Precuneus/posterior cingulate	2,252	−2, 52, 30 4, –50, 18	Positive
Left angular/middle occipital gyrus	658	−44, −76, 32 −48, −68, 30	Positive
Left superior frontal gyrus	500	−20, 32, 46	Positive
Right anterior insula	269	38, 16, 0 46, 16, –12 48, 14, –10	Positive
Bilateral thalamus	243	−8, −14, 8 10, –16, 6	Positive
Cluster 4 (posterior)			
Large cluster including: Ventromedial PFC Bilateral medial ventral striatum Bilateral amygdala Bilateral hippocampus Left central OFC Superior frontal gyrus	6,785	0, 30, –12 24, –2, −22 −2, 40, 2 −6, 6, –8 −2, 2, –12 14, 26, –10	Positive (all regions)
Precuneus/posterior cingulate	1,061	−4, −56, 20 −2, −52, 30 6, –52, 18 0, –36, 38 10, –56, 28	Positive
Left angular/middle temporal gyrus (PGp/PGa)	329	−48, −68, 32 −50, −66, 20	Positive
Right dorsal insula	238	38, 4, 10 42, –2, 4	Negative
Left putamen/left dorsal insula	148	−28, 4, 8 −42, −6, 8	None
Cluster 5 (central ventral)			
Large cluster including: Ventromedial PFC Posterior frontal pole/dorsomedial PFC Bilateral medial ventral striatum Left amygdala Left hippocampus	5,303	−2, 38, –16 −22, −14, −22 −2, 62, 4 8, 10, –10 8, 54, –8 −12, 42, 42	Positive (all regions)
Precuneus/posterior cingulate	1,009	−4, −56, 20 2, –54, 30 4, –56, 40	Positive
Right amygdala (LB/SF)/right hippocampus (CA)	540	22, –4, −20 28, –16, –20	Positive
Left central OFC/ventrolateral PFC	352	−36, 32, –16 −38, 36, –12	Positive
Left angular/middle occipital gyrus (PGp/PGa)	171	−42, −78, 30 −50, −66, 30 −44, −72, 24	Positive
Left middle temporal gyrus	121	−50, 4, –30	Positive
Cluster 6 (left sgACC)			
Large cluster including: Posterior ventromedial FL Bilateral caudate Bilateral medial VS Bilateral amygdala (LB) Right hippocampus	3,081	−16, 14, –14 8, 8, –12 14, 10, –14 8, 6, 2 16, –2, −2 24, –10, −20	Positive including: vmFL Caudate VS Amyg/HC
Thalamus (prefrontal/temporal‐connected region)	454	4, –18, 8 −4, −16, 6 −10, −16, 8 −10, −30, 8	None (positive but n.s.)

Abbreviations: Amyg, Amygdala; CA, cornu ammonis; HC, hippocampus; LB, laterobasal subregion; MACM, meta‐analytic connectivity modeling; OFC, orbitofrontal cortex; PFC, prefrontal cortex; rsfMRI, resting‐state fMRI; SF, superficial subregion.

Comparison of each cluster's MACM map was performed. First, in order to identify crucial regions that contributed to the parcellation, we contrasted each region with all of the other five regions (finding described in Table 3). Contrasting in a similar way rsfMRI maps largely corroborated these findings, insofar as all clusters aside from 4 and 6, and Cluster 2's parietal region, showed greater connectivity in the same region(s) as identified by the MACM contrasts (*p* < .001). In the case of Cluster 6, this effect was somewhat weaker (*p* < .05), but for Cluster 4, no difference in rsfMRI was seen in the putative hypothalamic region. Three of the more anterior regions (central dorsal and ventral, anterior) all showed preferential connectivity with areas of the DMN, including the PCC and SFG, although with slightly different emphasis in each case. In addition, the anterior region showed preferential connectivity with the left VLPFC. The three more posterior regions showed preferential connectivity with the insula, as well as subcortical regions such as the thalamus and basal ganglia.

**TABLE 3:** Regions with unique connectivity with each subregion, by finding the overlap of each cluster contrasted with all other clusters

Region	Unique clusters
Cluster 1	Right insula (32 voxels; 38, 21, 2)
Cluster 2	Left ventrolateral prefrontal cortex (13 voxels; −48, 29, −15) Left middle temporal gyrus (9 voxels: −57, 1, –20) Frontal pole/Fp2 (3 voxels: 6 64 10) Left inferior parietal lobule (PGa: 2 voxels; −55, −57, 28) Posterior cingulate cortex (2 voxels; 4, 47, 27)
Cluster 3	Left superior frontal gyrus (4 voxels; −18, 32, 40) Posterior cingulate cortex (2 voxels; −5, 47, 30)
Cluster 4	Possible hypothalamus (9 voxels; 4, −7, −8)
Cluster 5	Left superior frontal gyrus (28 voxels; −9, 43, 41) Left central orbitofrontal gyrus (13 voxels; −39, 39, −15)
Cluster 6	Thalamus (prefrontal/temporal‐connected regions: 223 voxels; −4, −18, 7) Right caudate (95 voxels; 8, 8, 5) Right pallidum (28 voxels; 20, 0, −6)

*Note*: Clusters 4 and 6, and Cluster 2 parietal were not strongly corroborated by a similar rsfMRI analysis (*p* < .001), but other regions were. Note that the hypothalamus region identified by Cluster 4 is on the very edge of the raw Cluster 4 MACM image, rather than being a distinct activation. Consequently, this may reflect an artifact of smoothing.

Abbreviations: MACM, meta‐analytic connectivity modeling; rsfMRI, resting‐state fMRI.

We also employed a similar method to examine rsfMRI functional connectivity, in which one seed was contrasted with all others to obtain regions where a given seed region was more positively correlated with target regions compared to the other regions. Cluster 1 revealed widespread connectivity, predominantly with “task‐positive” regions such as the lateral prefrontal cortex and regions of the parietal lobe (central executive network), the anterior insula and supplementary motor area (“salience network”), as well as the visual cortex, caudate, premotor cortex and regions of the cerebellum. Cluster 2 was positively connected to DMN regions, particularly the dorsomedial prefrontal cortex, posterior cingulate/precuneus, inferior parietal lobule, cerebellum, and temporal lobe. Cluster 3 was also strongly connected to similar DMN regions, but also more ventromedial frontal regions and, insula, striatum (caudate/putamen), hippocampus (but more weakly with the amygdala), thalamus and midbrain structures. Cluster 4 was also connected to DMN structures, but also medial temporal lobe regions (amygdala/hippocampus), somatosensory/motor cortex, posterior insula, and VLPFC. Cluster 5 structures were very similar to those identified in Cluster 4: direct contrast of Clusters 4 and 5 yielded greater insula connectivity for Clusters 4 than 5, and greater visual cortex and cerebellum connectivity for Cluster 5 than 4. Finally, Cluster 6 yielded similar central executive, salience and visual network regions to Cluster 1. Direct comparison of Clusters 1 and 6 suggested that Cluster 1 was better coupled to “task‐positive” networks than Cluster 6, and vice versa for Cluster 6 and the default mode regions.

### 3.3 Functional decoding

**FIGURE 4:**
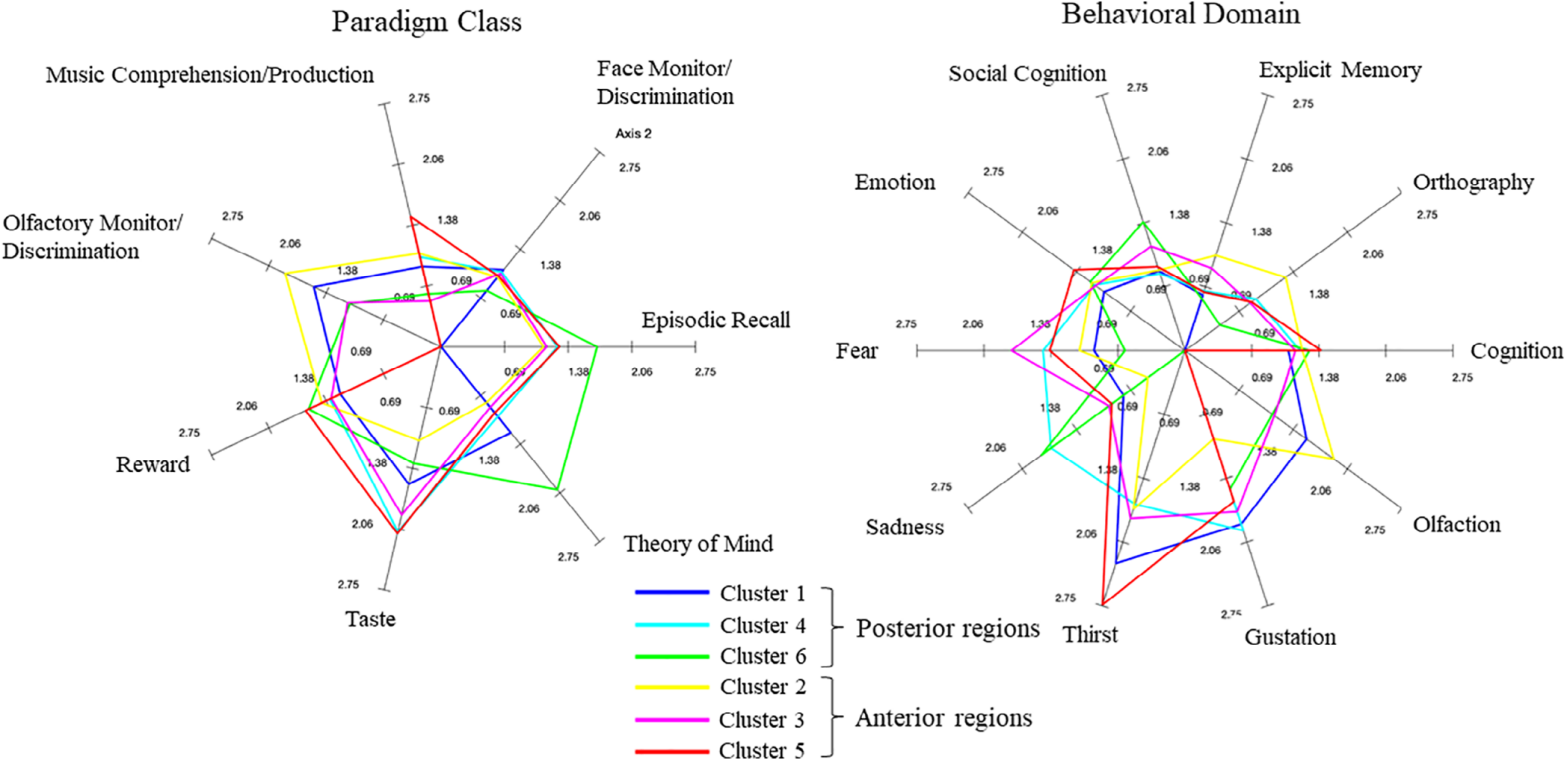
Spider plots describing forward inference (probability of activation given paradigm/domain) for each of the study types identified as significant for one or more subregion(s) (Table S1). Axes describe likelihood of activation, log‐transformed for display purposes

We also interrogated the BrainMap database to characterize the functional properties of the regions (Figure 4: fuller description of cluster comparisons is included in Tables S1 and S2). Emotion, cognition and reward were domains/paradigms that were most commonly represented across all regions, with relatively little preference for one region over any of the others in these dimensions, and numerous conjunctions between regions. Interestingly, while forward inference likelihoods were relatively well matched across relevant paradigms (Figure 4), cognition and reward showed much larger posterior probabilities of decoding paradigm/domain from an activation using reverse inference (~0.15–0.2) than other types of study (generally ~0.015–0.05). This might be related to a greater frequency of reward/cognition studies in the BrainMap database, which would be associated with a greater prior probability for these studies.

Gustation and taste paradigms were also quite widely represented, with perhaps Clusters 3, 4, and 5 showing a slight advantage. Thirst also modulated activity in regions 3 and 5. Evidence for selectivity was seen in other domains/paradigms, including olfaction/olfactory monitoring and discrimination, in which Cluster 2 was relatively selectively engaged. Together, this might suggest the presence of regional specialization of gustatory and olfactory systems in the vmFL. Although this preference was mostly qualitative rather than supported by clear dissociations, Cluster 2 did show significant preference for olfaction over Clusters 4 and 5.

Several candidate cognitive subdomains or paradigms were highlighted within the overall “cognition” domain: these included explicit memory (Cluster 2), episodic recall (Cluster 6), orthography (Cluster 2), social cognition (Cluster 3), face monitoring/discrimination (Cluster 3), theory of mind (Cluster 6), and music comprehension/production (Cluster 5). Finally, as well as reward paradigms, evidence for the representation of negative affect in the vmFL was obtained, insofar as sadness (Cluster 4) and fear (Clusters 3, 4, and 5) domains were also identified as capable of activating subregions of the vmFL.

## 4 DISCUSSION

In the present study, we aimed to identify distinct functional regions within the vmFL. We distinguished six subregions within a two‐stage parcellation process, in which a candidate vmFL was initially identified from surrounding areas (e.g., dorsal ACC, lateral/central OFC, and rostral prefrontal cortex) before it was subdivided further. The six clusters can be summarized as follows, moving in order from posterior to anterior: most posterior were separate left and right subgenual ACC regions, followed by a posterior region, two central regions (one dorsal and one ventral), and finally an anterior region (Figure 1). Correspondence between each of these regions with conventional anatomical labeling is discussed in detail in Section 3.1 Each region was, by definition, associated with differential MACM‐estimated connectivity, but these differences were also supported by distinct resting fMRI connectivity for each region. Moreover, some differences in the functional properties of the clusters were also observed, although in many cases similarities in these properties, particularly with respect to reward and emotion, were also pronounced.

The findings extend previous investigations using diffusion tensor imaging (Jackson, Bajada, Lambon Ralph, & Cloutman, 2019; Neubert, Mars, Sallet, & Rushworth, 2015), resting fMRI (Kahnt et al., 2012; Samara et al., 2017, Neubert et al., 2015), structural MRI (Liu, Qin, Qi, Jiang, & Yu, 2015) and value fMRI studies (Clithero & Rangel, 2014) which suggested that distinct neural regions could be identified within the human vmFL. Interestingly, all of these studies have yielded somewhat different findings, both from one another and the present work. In general, they have examined larger regions of the ventral or medial prefrontal cortex, and have parcellated the region at a lower resolution than we have here. To the best of our knowledge, our study is the first to employ a two‐stage parcellation technique, which may have provided greater capacity to distinguish particular subregions within the vmFL. In addition, prior research largely reflects anatomical or resting differences. However, there is evidence that functional clusters within in the vmFL might change following dopaminergic manipulations (Kahnt & Tobler, 2017), while other evidence suggests that, due to dynamic shifts in connectivity, functional clustering may differ based on task state (Iraji, Miller, Adali, & Calhoun, 2020; Salehi et al., 2020). Thus, our findings add to this literature by examining across a wide variety of tasks states within the BrainMap database. An exception within the prior vmFL studies is the study of Clithero and Rangel (2014), which involved a parcellation of coordinate maps—although these were restricted to studies of value. This study identified three clusters, one of which roughly reflected a frontal polar region. The remaining dorsal and ventral regions might approximately map onto our dorsal central and posterior/subgenual clusters, respectively. These latter regions were characterized by distinct patterns of functional connectivity, with the dorsal central region showing connectivity with the dorsal PCC, SFG, ventral striatum, and central OFC. Our findings are consistent with these findings insofar as similar regions are identified and play a role in distinguishing the different vmFL subregions (particularly SFG and PCC). The parcellation of the medial prefrontal cortex by Samara et al. also shows similarities with our findings (Samara et al., 2017), yielding around four regions which approximately overlap with our six subregions. This article is suggestive that a combination of rsfMRI and MACM may yield a “consensus” parcellation consisting of subregions defined jointly by both metrics, while examining differences between task states may provide further insight into the potential for a state‐independent parcellation which maps onto anatomical regions.

### 4.1 Comparison with other parcellation schemes: Technical considerations

The subregions we identified did not map cleanly onto two MNI‐based anatomical labeling templates of the vmFL (Henssen et al., 2016; Mackey & Petrides, 2014), with most of the CBP‐defined clusters mapping on to approximately 2–4 anatomical labels. Before considering reasons why this might be the case, it is important to note that a very anterior region identified by the initial parcellation (to obtain the vmFL cluster used for the main parcellation) overlapped quite clearly with a frontal pole region within JuBrain (Bludau et al., 2014; a parcellation of the frontal pole and overlap with anatomical regions has been reported in Ray et al., 2015). Previous studies have also shown a good correspondence between a CBP‐based parcellation of the amygdala and cytoarchitectonic definitions of the region (Bzdok et al., 2013). Thus, given that the CBP method has been shown to yield distinct functional clusters that are consistent with anatomical labels, we might consider reasons we did not demonstrate this in the present study. First, the clustering method is dependent on the functional connectivity of the region and not on other properties. Thus, it is possible that there are stark differences in functional or anatomical properties across the vmFL which are not reflected in MACM‐based functional connectivity. For example, activation of the basolateral amygdala may be reflected in activity both in the OFC and medial PFC (Lee et al., 2010; Logothetis & Wandell, 2004), despite the possibility that they decode or process this information input differently (Rudebeck, Mitz, Chacko, & Murray, 2013). Likewise, activity in separate vmFL regions might converge onto a single distal region, or two regions which are below the resolution where they can be differentiated. Furthermore, recent evidence has suggested finely “interdigitated” networks (Braga & Buckner, 2017), which may be reflected in a patchwork which might cut across the boundaries of discrete clusters.

Second, for a clear structure/function overlap to be seen, the paradigms employed must necessarily be highly selective for the process subserved by the particular subregion. However, the functional decoding analysis yielded examples of paradigms/domains which were related to the activation of several subregions. This suggests, at least, that many of the paradigms which make up the BrainMap database are not selective in this regard. Moreover, this may also be a reflection of the proposed “distributed coding” within the vmFL: specifically, the proposal that the region represents information via a population code which may be widely distributed across the region (Kahnt, Heinzle, Park, & Haynes, 2010; van Duuren et al., 2009). Third, different anatomical methods can provide different specifications of vmFL parcellation (Henssen et al., 2016)/(Mackey & Petrides, 2014), and it may require a combination of methods—including richer anatomical and functional characteristics—to characterize the regions more decisively (Eickhoff et al., 2018; Vogel et al., 2020).

Finally, although different methods may yield compatible information about different aspects of the structure, there are still potential caveats of the approach we used here. In particular, susceptibility artifacts in the vmFL are often present in conventional (EPI) sequences (but can be addressed: for example, (Weiskopf, Hutton, Josephs, & Deichmann, 2006). These artifacts would be expected to lead to both a loss of signal, and consequently false negatives, and also a misplacement of the signal due to distortion. Although researchers interested in the vmFL may choose an imaging protocol appropriately, this would potentially lead to some bias within the BrainMap database. However, this caveat is partly addressed by our use of resting fMRI to confirm the patterns of functional connectivity. This convergent approach also provides further validation of MACM as a method of examining functional connectivity (Smith et al., 2009).

One area of future investigation would be the potential for examining effects of between‐participant heterogeneity, particularly with respect to discontinuous variation (Chiavaras & Petrides, 2000). Our approach assumes the presence of discrete clusters within the vmFL which are broadly comparable across individuals, but it may be that substantial individual differences in the size or reach of an area could create transition zones which are not easy to classify. In the present study, Cluster 4 may be an example (Section 3.3).

Overall, functional connectivity provides a valuable window into the cross‐species homology of a given brain region as the limitations of functional characterization (e.g., paradigms which are underspecified for a particular construct; regions which act in concert with others to support a particular process) do not fully apply to connectivity—given that anatomical connectivity is contextually invariant, and that functional connectivity is constrained by anatomical connectivity. In addition, considerable information available from translational studies (discussed in the following) can provide particular insight into connectivity and inform interpretation of human studies.

### 4.2 Key distal, functionally connected regions that distinguish the subregions

One prominent axis of differentiation, using MACM, was the relationship of the three more anterior regions (Clusters 2, 3, and 5) with regions classically associated with the DMN (e.g., parietal and PCC) and the left VLPFC, versus the association of the posterior regions (Clusters 1, 4, and 6) with regions including the insula, thalamus, and basal ganglia. Within the anterior regions, the most anterior subregion (Cluster 2) was distinguished with respect to the temporal lobe (Petrides & Pandya, 2007; Saleem et al., 2008), parietal cortex (Barbas, 1993) and left VLPFC (Barbas & Pandya, 1989). The central clusters both connected to the SFG (Yeterian, Pandya, Tomaiuolo, & Petrides, 2012), but were differentiated by left OFC (central ventral: Cluster 3) and PCC (central dorsal: Cluster 5).

Posterior regions were divided on the basis of functional connectivity with the insula (right sgACC: Cluster 1), the striatum and thalamus (left sgACC: Cluster 6), and possibly the hypothalamus (posterior: Cluster 4). Although the voxels which distinguish Cluster 4 are approximately where the hypothalamus should be (Reppucci & Petrovich, 2016), it is difficult to rule out an artifactual effect of image smoothness in this case, as the distinguishing voxels were toward the edge of the MACM cluster centered on the seed region. A notable feature of these posterior subregions was the presence of hemispheric specialization between Clusters 1 and 6. This may relate to the hemispheric differentiation of connected subcortical regions. Despite the functionally significant connections between the hippocampal formation and amygdala with the vmFL (Price, 1999; Reppucci & Petrovich, 2016), neither structure played a significant role in distinguishing any of the subregions using MACM. Indeed, all clusters showed some evidence of MACM‐defined functional connectivity in one or both of the structures.

Notably, this differentiation with respect to posterior and anterior subregions was somewhat different when using rsfMRI. In this case, the more posterior regions tended to show greater relative connectivity with “task‐positive” regions including the central executive and salience networks. Consistent with the MACM analysis, the more anterior regions showed greater connectivity with the DMN. In addition, while the posterior subregions had shown strong functional connectivity with subcortical regions using MACM, Cluster 3 (central dorsal) now showed particularly strong connectivity across striatum (Heilbronner et al., 2016) and midbrain (Amat et al., 2005; Celada, Puig, Casanovas, Guillazo, & Artigas, 2001; Jo & Mizumori, 2016; Patton, Bizup, & Grace, 2013; Price, 1999).

In summary, the capacity of the MACM algorithm to differentiate subregions of the vmFL appeared to be largely based on different patterns of connectivity within the DMN, but also, for more posterior regions, the striatum, thalamus, and insula.

### 4.3 Implications for cross‐species homology

The question of cross‐species homology within the vmFL is complex and enduring, due to obvious anatomical differences between mammalian species in this region (Wise, 2008). Moreover, approaches to resolution are often strongly dependent on the method employed, be it anatomical (Heilbronner et al., 2016) or behavioral (Birrell & Brown, 2000). For example, the presence of connectivity with the ventral striatum (specifically the nucleus accumbens shell) might be indicative of an IL cortex‐like region (Gorelova & Yang, 1997; Heilbronner et al., 2016). Partially consistent with this, Clusters 1 and 6 both showed strong connectivity with the ventral striatum, with Cluster 6 being defined by its interactions with this region. Notably, Cluster 4 also overlapped with the OFC subregions of the anatomical atlases (area 14/Fo2), suggesting that it might be an OFC/IL transitional zone. Importantly, connectivity of the IL with the amygdala can be modulated by excitatory input (Patton et al., 2013), and it is notable that positive functional connectivity between the amygdala and these posterior subregions was seen using MACM but not strongly using rsfMRI.

Cluster 3 may be a potential homolog of the PL cortex. A salient feature of this region was its connectivity with the midbrain, striatum, and thalamus as assessed with rsfMRI, but not MACM. In addition, functional connectivity with the thalamus and insula (Vertes, 2004), and striatum (Gorelova & Yang, 1997; Sesack, Deutch, Roth, & Bunney, 1989) would support the attribution to the PL. Moreover, the region overlapped with areas 32 and 24ab in the SPM anatomy toolbox templates (Henssen et al., 2016; Palomero‐Gallagher et al., 2015). The region was also characterized by strong default mode connectivity, consistent with the implication of the PL in the rodent DMN (Hsu et al., 2016). Interestingly, rsfMRI analysis did not reveal strong positive connectivity with the amygdala despite connectivity with other subcortical structures, consistent with data suggesting that PL activation inhibits the amygdala (Gomes & Grace, 2017; Rosenkranz & Grace, 2002). MACM did, however, yield evidence of positive functional connectivity, but MACM might be expected to be less sensitive to rapid sequential effects. Specifically, the amygdala and vmFL might be concurrently activated by a paradigm, and while the vmFL might begin to inhibit the amygdala, the impact of this inhibition might not be reflected in the BOLD signal right away, allowing the hemodynamic response function to fit the stimulus‐locked BOLD activation in the amygdala.

Cluster 5 is a potential homolog of the medial OFC due to its relatively anterior and ventral location, primarily overlapping with areas 11/14 (Mackey & Petrides, 2014) and Fo (Henssen et al., 2016). This region showed distal connectivity with a number of areas that would be consistent with a medial OFC homolog, including the amygdala, hippocampal formation, and temporal lobe (Price, 1999; Saleem et al., 2008). It was also characterized by connectivity with the central OFC, suggesting that within‐OFC connectivity (Barbas & Pandya, 1989) may be functionally relevant. The region was also characterized by connectivity with primary visual regions, at least in comparison with Clusters 3 and 4. It is likely that visual input would reach the medial OFC indirectly, via the temporal lobe (Rolls, 2004), connections which are thought to be crucial for visual perception (Bar et al., 2006).

As the most anterior region, Cluster 2 is perhaps most likely not to have a direct rodent homolog, and perhaps be characterized in terms of Brodmann area 10 (Ongur et al., 2003). As such, it may also be most likely to be eulaminate/granular (Barbas & Garcia‐Cabezas, 2016), as opposed to agranular or dysgranular like the more posterior vmFL regions we describe (Ongur et al., 2003). It showed functional connectivity with more advanced cortical areas such as the frontal pole and left ventrolateral cortex, both of which have shown evolutionary development in humans compared to rodents (Wise, 2008). However, it was partially overlapping with Mackay and Petrides' (Mackey & Petrides, 2014) areas 11 and 14, but also with Henssen et al.'s Fp region (Henssen et al., 2016).

In summary, the data provide some preliminary suggestions of potential, discrete homologs of the rodent IL/PL subregions and medial OFC in the human brain, as well as a more rostral region that might not be reflected in the rodent frontal lobe. These subregions might provide a basis for more detailed future investigations, including characterization of structure/function relationships in this region.

### 4.4 Clinical relevance and functional decoding

The vmFL is a critical locus of interest for understanding the symptoms of psychiatric disorders, particularly those associated with disturbances of emotional or social behavior (Hiser & Koenigs, 2018). Recent evidence has extended this to forms of pain, where it may play an inhibitory role (Leaver et al., 2011; Rauschecker, May, Maudoux, & Ploner, 2015). Our primary interest in differentiation of function within the vmFL has arisen from studies of mood disorders, which has been frequently associated with functional abnormalities within the subgenual cingulate cortex (Drevets, Price, & Furey, 2008). From a translational perspective, work by Maier and others has implicated a potential homolog of the vmFL in rodents as underlying the effects of uncontrollable stress and mitigating effects of behavioral control (Amat et al., 2005), a potential rodent model of major depression or posttraumatic stress disorder. More recent evidence suggested that these effects might be localized to PL regions (Christianson et al., 2014)—consistent with its role in instrumental control (Killcross & Coutureau, 2003)—whereas the subgenual region identified in depressed humans is typically thought be homolog of the IL cortex (Rudebeck et al., 2014). Our findings may contribute to this body of work by suggesting the presence of differentiation of function within the vmFL in the human brain (McNamee, Rangel, & O'Doherty, 2013) at a resolution that is relevant for the precise localization necessary for designing interventions for mood disorders (Mayberg et al., 2005). For example, Cluster 3, the potential homolog of PL which we identified here, was characterized by rsfMRI functional connectivity with the midbrain. This finding may be relevant for understanding the effect of uncontrollable stress on the dorsal raphe nucleus via regulation by the rodent PL/IL (Amat et al., 2005). In general, examination of vmFL functional connectivity in mood and anxiety disorders has employed a variety of methods and seed regions of interest, and our findings may help to provide tools to integrate some of these investigations. For example, our previous work suggests that seed‐based analysis which uses distinct subregions of larger neural structures can be an effective approach to identifying relevant individual differences (Chase et al., 2017). Further examination of the consistency of functional differentiation across illness and health, as well as across task conditions or mood state, remains to be determined.

In the present work, functional decoding of the vmFL provided independent confirmation of relevance for psychiatric disorders—particularly disorders involving deficits of emotional regulation and reward function such as mood disorders, given emotion (Roy, Shohamy, & Wager, 2012) and reward studies frequently activated the region. The region was also associated with cognition, perhaps via the frequent identification of task‐related deactivations or contrasts with reduced attentional demands (K. C. Fox, Spreng, Ellamil, Andrews‐Hanna, & Christoff, 2015). These neural dynamics might also be relevant for alternations in cognitive function in psychiatric disorders (Farruggia, Laird, & Mattfield, 2020). The capacity to decode paradigms/domains from vmFL activations was modest, but was markedly larger for reward and cognition than other types of study. This may be due to the larger proportion of these studies in the BrainMap database, which would make the posterior probabilities larger. While evidence of functional specialization within the vmFL was generally not strong, there was a suggestion of a spatial dissociation of gustatory versus olfactory processing (Rolls & Baylis, 1994), insofar as the anterior subregion (Cluster 2) was most strongly related to olfaction, whereas more posterior subregions (including 1, 4, and 5) were most strongly related to gustation. Together, the findings are broadly consistent with models of the vmFL which emphasize diversity of function via the representation of predictive models of the environment (Barrett & Simmons, 2015; Niv, 2019).

Within the present data set, one notable feature of the functional connectivity profiles of the different regions was that there was evidence that more posterior regions (Clusters 1, 6, and 4) showed more discrepant connectivity between MACM and rsfMRI. For example, voxels which uniquely characterized Clusters 4 and 6 using MACM did not show strong functional connectivity with Clusters 4 and 6 using rsfMRI. In addition, these posterior regions showed relatively higher rsfMRI functional connectivity with task positive than DMN regions, which contrasted somewhat with Cluster 4's MACM map. Recent theoretical work suggests that IL and PL cortices are in competition, with the IL mediating inflexible stimulus–response learning, and the PL underling flexible use of contextual cues to optimize task performance (Sharpe & Killcross, 2018). Given the novelty of the fMRI scanner and the tasks that participants are often asked to perform, this model might suggest that the PL‐homolog in humans might dominate, and show stronger and perhaps therefore more consistent functional connectivity patterns. By contrast, activation of the IL‐homolog might only be evident after over‐training and higher scanner familiarity. Moreover, IL activity might be (rapidly) followed by more “task‐positive” network activity, as individuals seek to overcome prepotent biases, should they become engaged. In this case, positive correlations between IL and task‐positive networks might be seen, given the sluggish nature of the BOLD response (Buxton, Uludag, Dubowitz, & Liu, 2004). Overall, the present findings suggest the potential to test this model empirically using functional neuroimaging data.

### 4.5 Summary

In the present work, we aimed to identify separate subregions of the vmFL, consistent with prior suggestions of differentiation in experimental animals and potential functional differences seen in humans. Using MACM‐based functional connectivity parcellation in two steps, we found evidence for a six subregion solution. The subregions included two unilateral subgenual cingulate regions, a posterior region, a ventral and dorsal central region and an anterior region. These regions were characterized by different patterns of within‐DMN and subcortical functional connectivity (using MACM and resting fMRI), and showed suggestions of functional differences. Together, the findings are consistent with the proposal that the vmFL may be divided into functionally independent subregions. Future work might aim to clarify differences between the derived functional subregions and previous observations of anatomical differentiation within the vmFL.

## CONFLICT OF INTEREST

None of the authors declare any financial or other conflicts of interest that might have biased the work.

## Supporting information


**Data S1**. Supplementary Information

## Data Availability

Data used in the study include the BrainMap database and the NKI/Rockland sample which are publically accessible. The derived clusters will be uploaded to the ANIMA database (http://anima.fz‐juelich.de/).
