# Supplementary material for: Functional differentiation in the human ventromedial frontal lobe: A data‐driven parcellation
Source: Hum Brain Mapp. 2020 Apr 21;41(12):3266–83. doi: 10.1002/hbm.25014 (PMC7375078; doi:10.1002/hbm.25014)
Supplement: Supplementary file 1 — Data S1. Supplementary Information [file HBM-41-3266-s001.docx]

Supplementary Information

| *Domains* | Cluster 1 | Cluster 2 | Cluster 3 | Cluster 4 | Cluster 5 | Cluster 6 |
| --- | --- | --- | --- | --- | --- | --- |
| Gustation | A/D; D/A | A/D | A/D | A/D; D/A | A/D; D/A | - |
| Cognition | A/D | A/D; D/A | A/D; D/A | A/D; D/A | A/D; D/A | A/D; D/A |
| Emotion | A/D | D/A | A/D; D/A | A/D; D/A | A/D; D/A | A/D; D/A |
| Olfaction |  | A/D; D/A |  |  |  |  |
| Orthography |  | A/D; D/A |  |  |  |  |
| Explicit Memory |  | A/D; D/A |  |  |  |  |
| Thirst |  |  | A/D; D/A |  | A/D; D/A |  |
| Fear |  |  | A/D; D/A | A/D | A/D |  |
| Social Cognition |  |  | A/D; D/A |  |  |  |
| Sadness |  |  |  | A/D; D/A |  |  |
| *Paradigms* |  |  |  |  |  |  |
| Reward | A/P | A/P; P/A | A/P; P/A | A/P; P/A | A/P; P/A | A/P; P/A |
| Olfactory Monitoring, Discrimination |  | A/P; P/A |  |  |  |  |
| Taste |  |  | A/P; P/A | A/P; P/A | A/P; P/A |  |
| Face monitoring, discrimination |  |  | A/P |  |  |  |
| Music Comprehension/Production |  |  |  |  | A/P |  |
| Theory of Mind |  |  |  |  |  | A/P; P/A |
| Episodic recall |  |  |  |  |  | A/P; P/A |

Table 4: Functional decoding analysis. Presence of significant (FDR corrected) forward inference (activation/paradigm: A/P; activation/domain: A/D) or reverse inference (paradigm/activation: P/A; domain/activation: D/A) of a given psychological paradigm or domain for each cluster.

| Domain | Regions pA/D | Regions pD/A | Conjunctions pA/D | Conjunctions pD/A | Differences pA/D | Differences pD/A |
| --- | --- | --- | --- | --- | --- | --- |
| Gustation | 1,2,3,4,5 | 1,4,5 | 1&3, 1&4, 1&5, 3&4, 3&5, 4&5 | 1&3, 1&4, 3&4, |  |  |
| Cognition | 1,2,3,4,5,6 | 2,3,4,5,6 | 2&3, 2&4, 2&5, 2&6, 3&5, 3&6, 4&5, 4&6, 5&6 | 2&3, 2&4, 2&5, 2&6, 3&4, 3&5, 3&6, 4&5, 4&6, 5&6 | 3>5 |  |
| Emotion | 1,3,4,5,6 | 2,3,4,5,6 | 1&5, 1&6, 2&3, 2&4, 2&5, 2&6, 3&5, 3&6, 4&5, 4&6, 5&6 | 2&3, 2&4, 2&5, 2&6, 3&4, 3&5, 3&6, 4&5, 4&6, 5&6 | 5>1/2/4  3>5 | 1>5  3/4>5 |
| Olfaction | 2 | 2 |  |  | 2 > 4/5  3>4/5 |  |
| Orthography | 2 | 2 |  |  | 2/3/4>1 |  |
| Explicit Memory | 2 | 2 |  |  | 2>5 |  |
| Thirst | 3,5 | 3,5 |  | 3&5 | 3,5>6 |  |
| Fear | 3,4,5 | 3 | 3&4 |  | 3>2/6  4>6 |  |
| Social Cognition | 3 | 3 |  |  |  |  |
| Sadness | 4 | 4 |  |  | 4>1/2/6  3>2 |  |
| Baserate |  |  |  |  | 3>4/5/6  3/4>2  2/4>5  2/3/4/5>1  1/4/5>6 |  |
| Phonology |  |  |  |  | 3>1/6 |  |
| Imagination |  |  |  |  | 3>1/6 |  |
| Memory |  |  |  |  | 3>1/5 |  |
| Anxiety |  |  |  |  | 3>1/4/6 |  |
| Sexuality |  |  |  |  | 4>1/2/6  3>2/6 |  |
| Working memory |  |  |  |  | 1>6 |  |
| Inhibition |  |  |  |  | 3>2 |  |
| Somatic cognition |  |  |  |  | 3>2,4,5,6 |  |
| Attention |  |  |  |  | 3,5>2 |  |
| Action Execution |  |  |  |  | 2>4 |  |
| Music |  |  |  |  | 4>3/6 |  |
| Disgust |  |  |  |  | 3>4/5/6 |  |
| Somesthesis |  |  |  |  | 3>5 |  |
| Shape perception |  |  |  |  | 3/4 > 6 |  |
| Happiness |  |  |  |  | 3 > 6 |  |

Supplementary Table 1: Cluster activations, and significant (FDR) comparisons (</>) and conjunctions (&) between clusters, in terms of their activation probabilities relating to different psychological domains.

| Paradigm | Regions pA/P | Regions pP/A | Conjunctions pA/D | Conjunctions pD/A | Differences pA/P | Differences pP/A |
| --- | --- | --- | --- | --- | --- | --- |
| Reward | 1,2,3,4,5,6 | 2,3,4,5,6 | 1&2, 1&3, 1&4, 1&5, 1&6, 2&3, 2&4, 2&5, 2&6, 3&4, 3&5, 3&6, 4&5, 4&6, 5&6 | 1&2, 1&4, 1&5, 1&6, 2&3, 2&4, 2&5, 2&6, 3&4, 3&5, 3&6, 4&5, 4&6, 5&6 | 5>1  3>5 |  |
| Olfactory Monitoring, Discrimination | 2 | 2 |  |  | 2>4/5  3>4/5 |  |
| Taste | 3,4,5 | 3,4,5 | 3&4, 3&5, 4&5 | 3&4, 3&5, 4&5 | 5>2  3>6 |  |
| Face monitoring, discrimination | 3 |  |  |  |  |  |
| Music Comprehension/Production |  | 5 |  |  | 5>3 | 3>5 |
| Theory of Mind | 6 | 6 |  |  |  |  |
| Episodic recall | 6 | 6 |  |  | 6/5/4/3/2>1 |  |
| Baserate |  |  |  |  | 5/4/3/2>1  1/2/3/5>6  2>5  3/4>2  4>3/5/6 |  |
| Delayed match to sample |  |  |  |  | 1<2/3/5 | 1>3 |
| Imagined objects, scenes |  |  |  |  | 3/2>1 |  |
| Film viewing |  |  |  |  | 1/4>3  4>6 | 1/4>3 |
| Classical conditioning |  |  |  |  | 3>1/6 |  |
| Word generation (covert) |  |  |  |  | 3>1/5 |  |
| Orthographic Discrimination |  |  |  |  | 3>1/4/5 |  |
| Rest |  |  |  |  | 3>1/2/5/6 |  |
| Delay Discounting |  |  |  |  | 3>1/2 |  |
| Encoding |  |  |  |  | 4>1/6  2>6  3>6 |  |
| Stroop |  |  |  |  | 3>2/5/6 |  |
| Tone monitor/  discrimination |  |  |  |  | 3>2 |  |
| n-back |  |  |  |  | 2/3/4>6 |  |
| Pain Monitor / Discrimination |  |  |  |  | 3>5 |  |
| Visuospatial Attention |  |  |  |  | 3/4/5>6 |  |
| Paired Associated Recall |  |  |  |  | 3>6 |  |
| WCST |  |  |  |  | 3>6 |  |
| Reading (overt) |  |  |  |  | 4>5 |  |

Supplementary Table 2: Cluster activations, and comparisons/conjunctions between clusters, in terms of their activation probabilities relating to different paradigms.


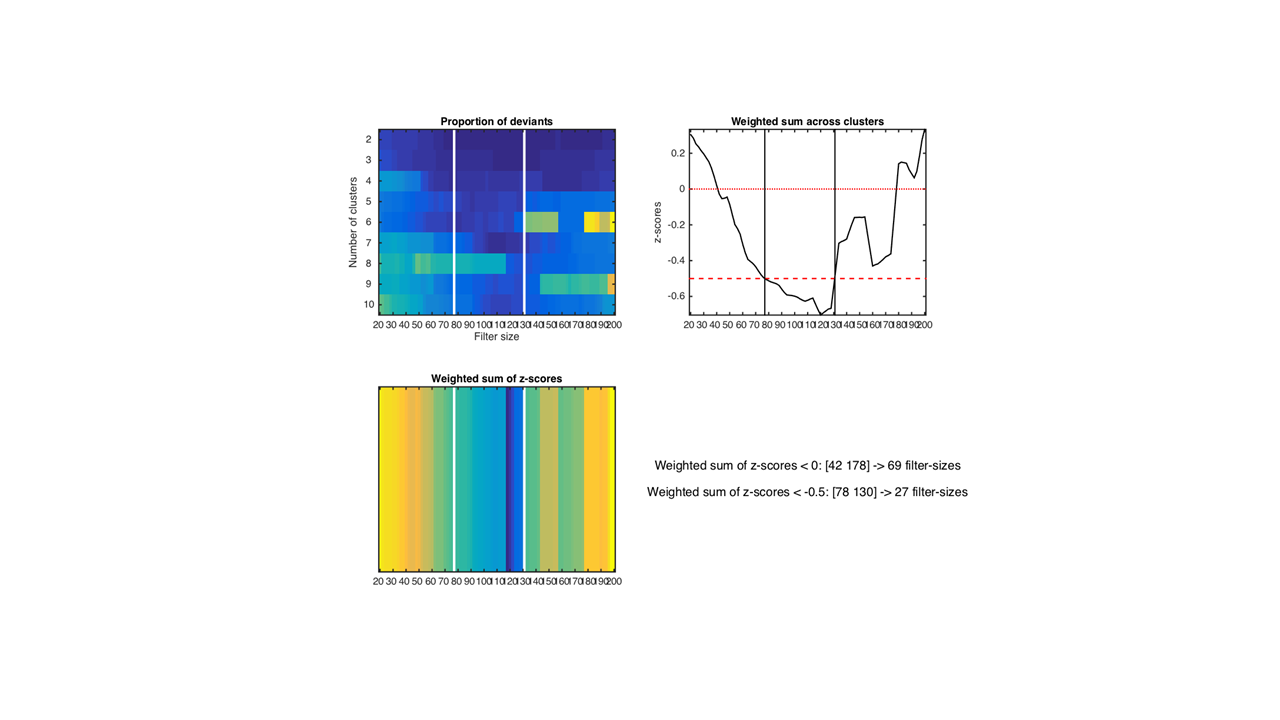


Supplementary Figure 1: Figure describes analysis of proportion of deviants used to determine filter range.


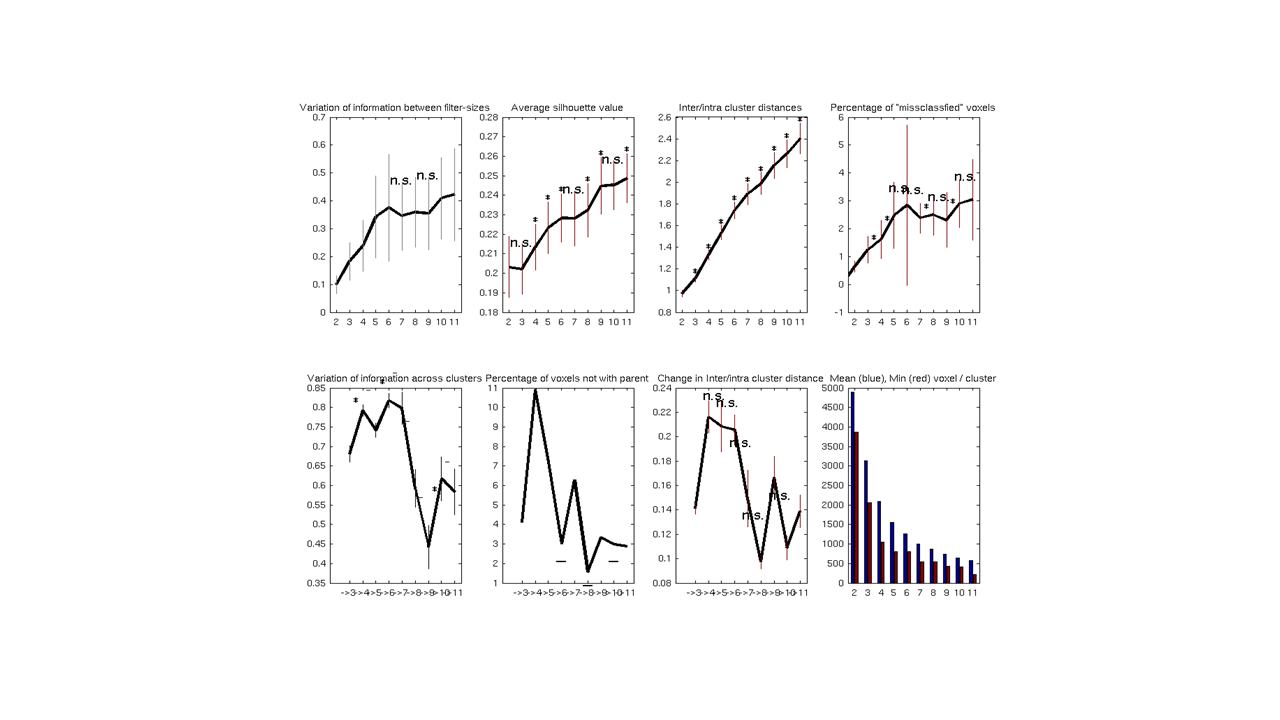


Supplementary Figure 2: Fit indices for first parcellation, including variation of information between filter sizes, average silhouette value, inter/intra cluster distances, percentage of misclassified voxels, variation of information across clusters, percentage of voxels not with parent and change in inter/intra cluster distance. In addition, the voxel size of the smallest and largest cluster for a given cluster number is included. Stars (*) represent a significant difference associated with a cluster transition (i.e. n to n+1) or associated with a cluster (n), and n.s. reflects no significant difference.


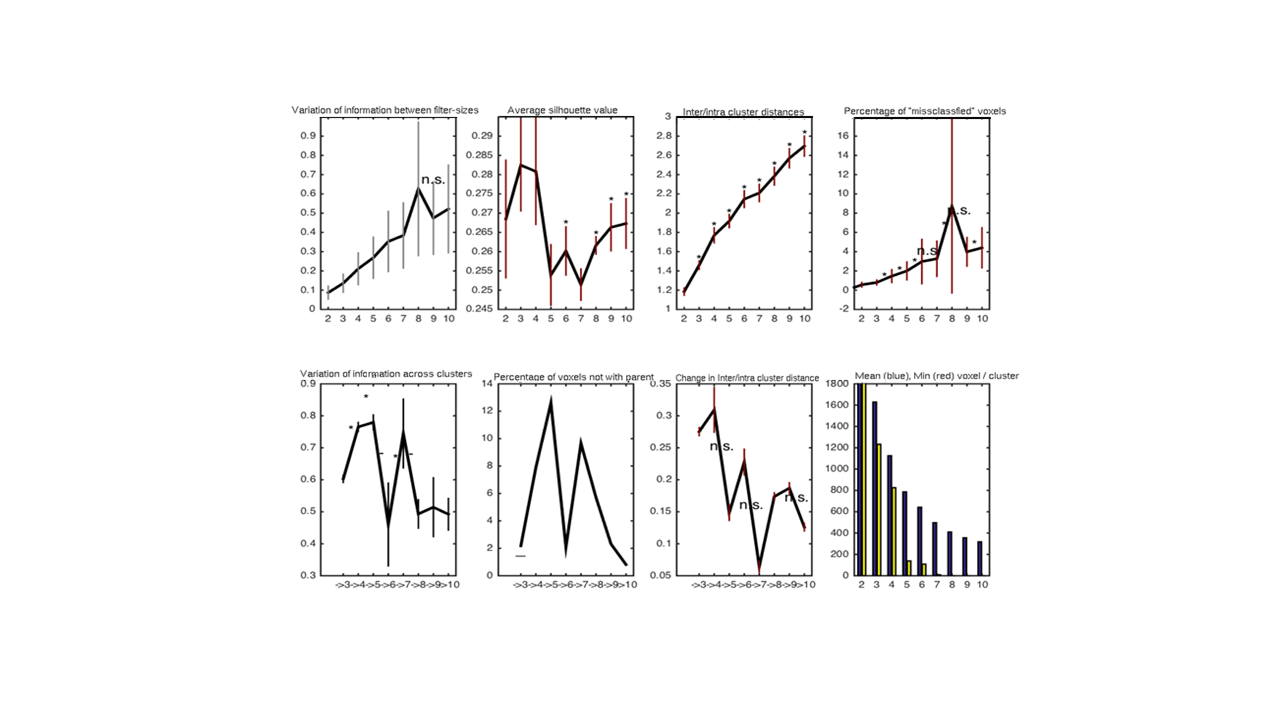


Supplementary Figure 3: Fit indices for second parcellation, with fit indices the same as Figure 2.


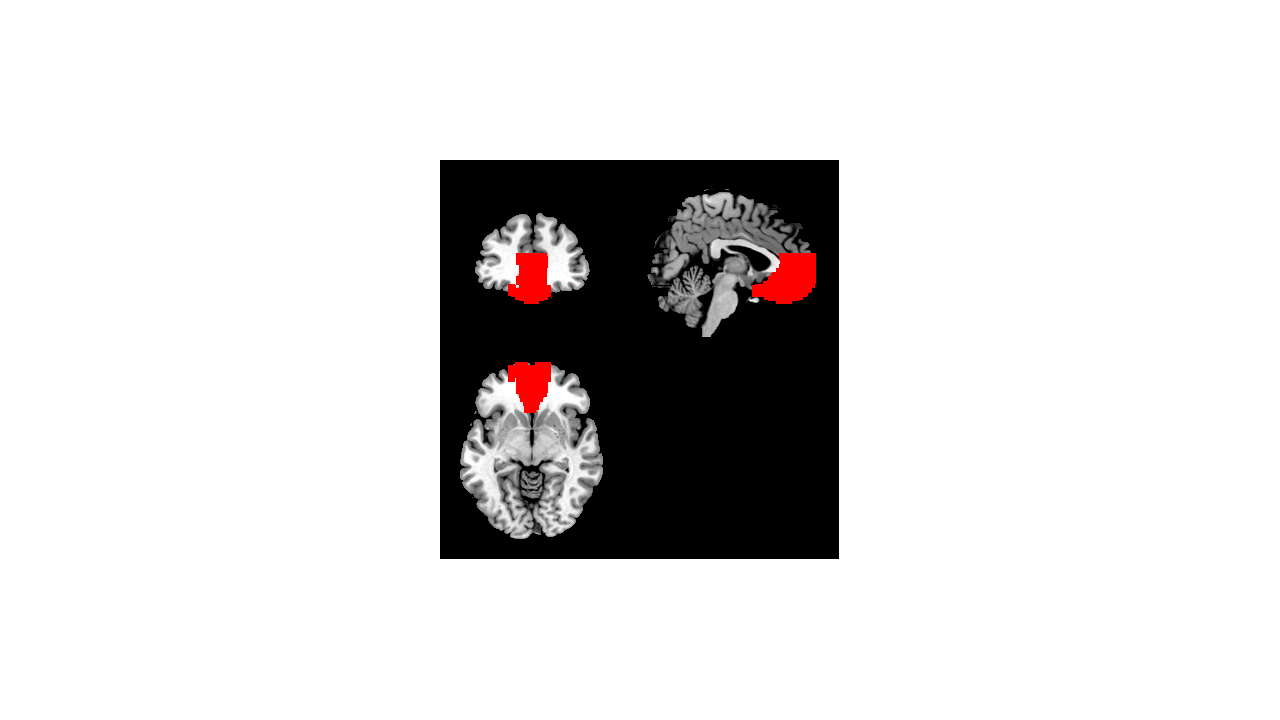


Supplementary Figure 4: Original vmFL ROI, used for the first parcellation.


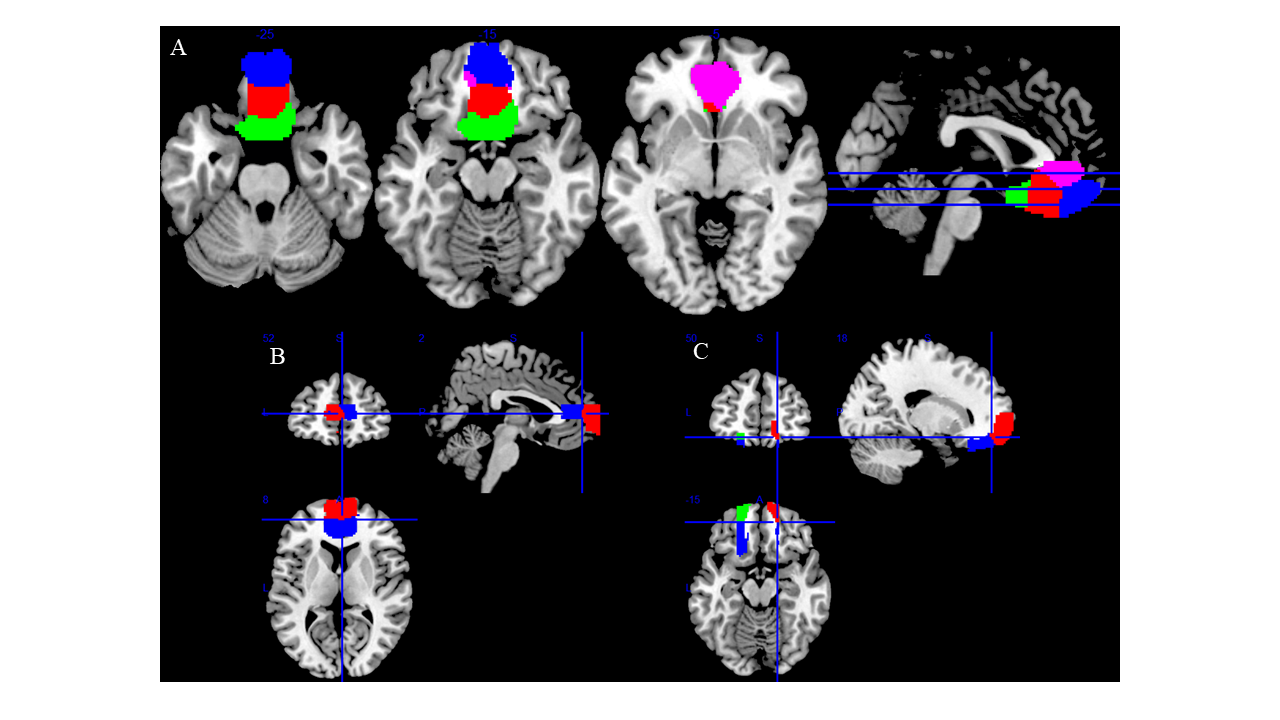


Supplementary Figure 5: Clusters derived from the first parcellation. A) clusters 2,4,7 and 8 – these formed the basis for the second parcellation; the following clusters were ignored for further analysis B) clusters 1 and 3 – unresolved at the dorsal edge; C) clusters 5, 6 and 9 – unresolved at the lateral edge.


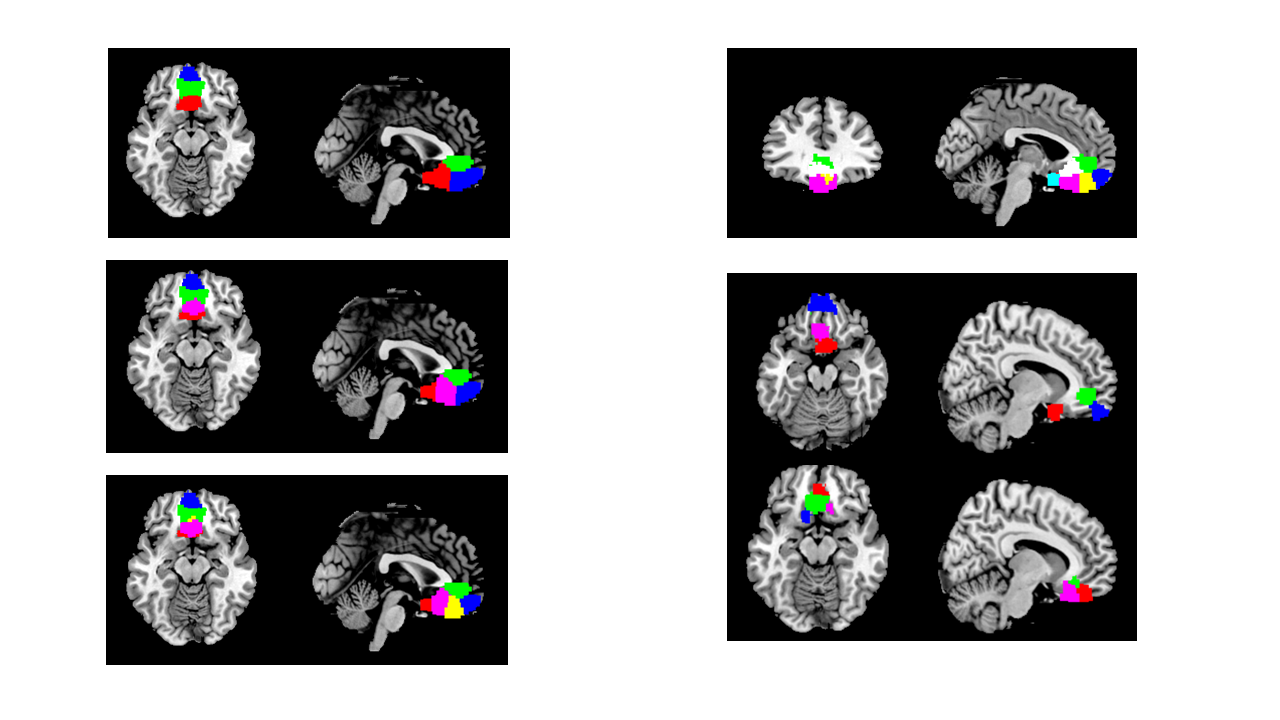


Supplementary Figure 6: Clusters obtained from the non-selected parcellations: 3-, 4-, 5-cluster solutions (top to bottom: left); and 7-, 8-cluster solutions (right).
